# Association between Breastfeeding Duration and Long-Term Midwifery-Led Support and Psychosocial Support: Outcomes from a Greek Non-Randomized Controlled Perinatal Health Intervention

**DOI:** 10.3390/ijerph18041988

**Published:** 2021-02-18

**Authors:** Maria Dagla, Irina Mrvoljak-Theodoropoulou, Marilena Vogiatzoglou, Anastasia Giamalidou, Eleni Tsolaridou, Marianna Mavrou, Calliope Dagla, Evangelia Antoniou

**Affiliations:** 1 Day Center for the Care of the Mental Health of Women (Perinatal Mental Health Disorders), Non-Profit Organization “FAINARETI”, 17121 Athens, Greece; imrvoljak@hotmail.com (I.M.-T.); marielena.vogiatzoglou@gmail.com (M.V.); nasiamami@gmail.com (A.G.); etsolaridou@uniwa.gr (E.T.); aebmc19021@uniwa.gr (M.M.); kelkeldag@gmail.com (C.D.); lilanton@uniwa.gr (E.A.); 2 Department of Midwifery, University of West Attica, 12243 Athens, Greece

**Keywords:** breastfeeding, mental health, intervention, mental health disorders

## Abstract

Background: This study investigates if a non-randomized controlled perinatal health intervention which offers (a) long-term midwife-led breastfeeding support and (b) psychosocial support of women, is associated with the initiation, exclusivity and duration of breastfeeding. Methods: A sample of 1080 women who attended a 12-month intervention before and after childbirth, during a five-year period (January 2014–January 2019) in a primary mental health care setting in Greece, was examined. Multiple analyses of variance and logistic regression analysis were conducted. Results: The vast majority of women (96.3%) initiated either exclusive breastfeeding (only breast milk) (70.7%) or any breastfeeding (with or without formula or other type of food/drink) (25.6%). At the end of the 6th month postpartum, almost half of the women (44.3%) breastfed exclusively. A greater (quantitatively) midwifery-led support to mothers seemed to correlate with increased chance of exclusive breastfeeding at the end of the 6th month postpartum (*p* = 0.034), and with longer any breastfeeding duration (*p* = 0.015). The absence of pathological mental health symptoms and of need for receiving long-term psychotherapy were associated with the longer duration of any breastfeeding (*p* = 0.029 and *p* = 0.013 respectively). Conclusions: Continuous long-term midwife-led education and support, and maternal mental well-being are associated with increased exclusive and any breastfeeding duration.

## 1. Introduction

Breastfeeding has been linked to the better health and wellbeing of women and their children [1,2,3,4,5], and, at the same time, is considered as one of women’s reproductive rights. Every woman has the right to make independent decisions about her body, including whether to breastfeed and the duration of breastfeeding. Every society has an obligation to ensure the minimum conditions that will allow every woman to freely and autonomously exercise her right to breastfeed. According to the United Nations Children’s Fund (UNICEF) and the World Health Organization (WHO) [6], every society should invest in breastfeeding support programs and policies that put women’s rights, dignity and choice at the center, because “Supporting a woman’s right to breastfeed is a measure of gender equality—and building a breastfeeding-friendly society is everyone’s responsibility” [6]. 

Globally, several evidence-based strategies have been proposed [7] and breastfeeding interventions have been implemented that support mothers breastfeeding exclusively and for a long period [8]. According to systematic reviews and meta-analyses, breastfeeding support interventions that focus on prenatal/antenatal education and continuous long-term care reinforce the intention of women to breastfeed antenatally; such interventions are supportive of the duration and exclusivity of breastfeeding, and are characterized as effective [9,10,11,12,13,14,15,16,17]. At the same time, as it has been proven that the initiation and duration of breastfeeding is negatively affected by the existence of mental health disorders in women [18,19,20,21], the international scientific community has recognized the need to implement interventions aimed at supporting women’s mental health before and after childbirth, in order to prevent the premature cessation of breastfeeding [22,23,24]. Women with anxiety disorders or depressive mood seem to experience difficulty with breastfeeding and often do not breastfeed exclusively or do not breastfeed for a long time [20,25]. Thus, the investigation into the role of breastfeeding interventions that support women who experience symptoms of mental health disorders during perinatal period in the improvement of breastfeeding initiation and continuation is of utmost importance.

In Greece, such a breastfeeding support intervention has been integrated into a perinatal mental health intervention, which offers midwife-led breastfeeding education, counselling and long-term support as well as psychosocial support, and aims to promote women’s mental health during the perinatal period. This intervention is implemented in a primary mental health center, namely the “Day Center for the Care of the Mental Health of Women (Perinatal Mental Health Disorders)”. This Day Center was created in 2009 by FAINARETI, a Non-Profit Organization that aspires to develop actions, through specialized midwifery and psychosocial interventions, to improve perinatal care in Greece. It is funded and supervised by the Greek Ministry of Health and all its services are provided free of charge. It is the first and only specialized Day Center in Greece that aims at the prevention, early detection and treatment of perinatal mental health disorders. 

The innovation of the Day Center’s program lies in (a) the interdisciplinary cooperation (among midwives, psychologists and psychiatrists), (b) the early education and awareness raising of women and their partners (on issues related to mental health, such as breastfeeding, etc.), (c) the early and continuous screening of all pregnant women/new mothers and their partners and also the timely treatment of symptoms of perinatal mental health disorders, and (d) the continuous monitoring and support of women and their partners (from the beginning of pregnancy until the end of the 1st year postpartum) and not only for people who suffer, but also for those who are at high risk (or not) of developing a mental health disorder.

The investigation of breastfeeding initiation and duration in such a context, as that of the Day Center, which educates, advises and supports women, and, at the same time, takes special care of their mental health, is of interest and importance. The role of such breastfeeding interventions is worth exploring, especially in countries with unsatisfactory breastfeeding rates, including Greece. Although in the last decade in Greece, important steps have been taken at the institutional and social level that have brought positive changes in the epidemiological indicators of breastfeeding [26,27], the reality is still far from the recommendations of international organizations [27,28], since the prevalence of exclusive breastfeeding at the end of the 6th month remain at a very low level (<1%) [27]. The investigation of the factors associated with the exclusivity and duration of breastfeeding has a particular value, as it offers new knowledge which would be useful for the designing of new strategies and interventions that act to support and promote breastfeeding. 

The aim of this study is (a) to investigate if a perinatal health intervention (12 month) that combines (i) continuous long-term midwife-led breastfeeding support (antenatal education and continuous counseling/lactation support), with (ii) the psychosocial support of women (early and continuous mental health screening, psychosocial/psychiatric care—when necessary—and monitoring), is associated with the initiation and duration of exclusive and any breastfeeding and (b) to identify the factors that are related with the duration of exclusive and/or any breastfeeding. 

## 2. Materials and Methods 

### 2.1. Study Population

This study analyzed data that emerged from the operation of the Day Center in Athens (Greece) during a five-year period (January 2014–January 2019). In order for a woman to attend the Day Center’s program, she must: (a) be pregnant, (b) be over 18 years old, (c) not use drugs, and (d) not be in need of hospitalization due to a mental health problem. This analysis includes data from all the women that took part in the program during that time period, except (a) those who did not participate in the antenatal breastfeeding education program, but joined after giving birth, (b) those who did not complete the full intervention program and for any reason discontinued their participation before the end of the 1st year postpartum and (c) those for whom there were insufficient recorded data. The final sample of the study consisted of 1080 women.

### 2.2. Procedure

#### The Midwifery and Psychosocial Intervention of the Day Center

Initially, pregnant women had a first contact with the perinatal health intervention and the services it offers through the website of the FAINARETI Organization (https://www.fainareti.gr/en/ (accessed on 20 November 2020)). The women could then express their interest and apply for participation in the program through a telephone intake process. An appointment was then scheduled to inform the pregnant woman and her partner about the purpose of the program and what it comprised. At the end of the briefing, consent to participate in the program was obtained. The next appointment included a complete medical, psycho-emotional and social history taken by a midwife, through which the individual needs of each person were assessed. Based on those needs, a specialized midwifery and psychosocial intervention program was determined that was completed at the end of the 1st year postpartum.

**Figure 1:**
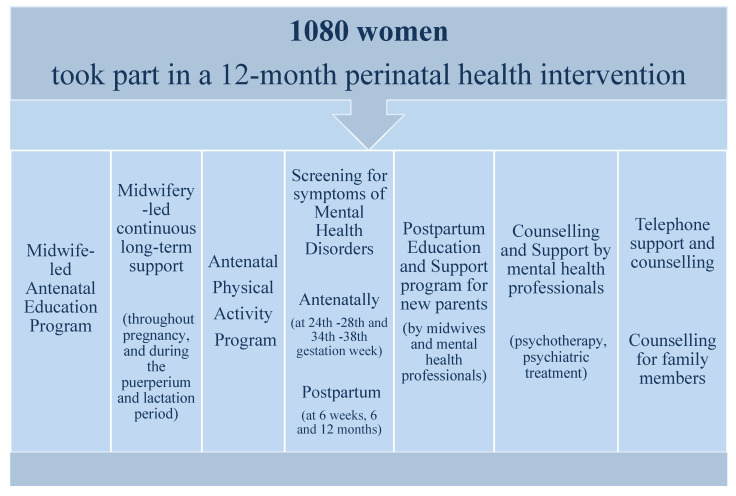
The Perinatal Health Intervention of the Day Center.

The services offered by the Day Center’s perinatal health intervention (Figure 1) included: (a) A midwife-led antenatal education and preparation for labor and parenthood program (8–12 three-hour group sessions or 4–5 two-hour individual sessions) that discussed issues concerning the pregnancy, fetal development, the pregnant woman’s preparation for delivery, labor pain relief, breastfeeding, the emotional changes that occur during the postpartum period, baby care-taking, behavior and adjustment of the baby and the partner’s role in the perinatal period. Each woman and her partner were able to attend up to 3 three-hour educational sessions pertaining to breastfeeding. (b) Antenatal and postpartum screenings for symptoms of mental health disorders of each woman and her partner. Screening took place before the start of the antenatal education and preparation for labor and parenthood program (approximately between the 24th and 28th gestation week), at its completion (approximately between the 34th and 38th gestation week), at 6 weeks postpartum and at 6 and 12 months postpartum. (c) An antenatal physical activity program conducted by a specialized physical education teacher (group and individual sessions). (d) Midwife-led counselling and support throughout pregnancy, and during the puerperium and lactation period (monitoring of the mother and her neonate and breastfeeding/lactation support by specialized midwives). During the first 3–4 days after delivery, when mother and newborn were hospitalized, every mother had access to midwifery-led counseling and support via telephone calls. Then, throughout the puerperium and lactation period, midwifery-led counseling and support continued either through mother-infant visits to the Day Center, or via telephone calls. (e) Counselling and support by mental health professionals (psychological assessment, monitoring and psychotherapy, psychiatric assessment and treatment) for both women and their partners, if it was necessary, during pregnancy and 12 months postpartum. (f) A postpartum education and support program for new parents that took place in the 3rd, 6th and 12th month after birth. This included the provision of counselling and support by midwives and mental health professionals, aiming at the adaptation of the parents to their parental role, the support of breastfeeding and at solving problems related to the baby’s behavior (e.g., sleep, introduction of solid foods, etc.). (g) Telephone support and counselling through a Pan-Hellenic helpline staffed by midwives and mental health specialists. (h) Counselling by a midwife and/or a mental health professional for family members or other people that could support the women and their partners. Oral and written consents of all the pregnant women who participated in the Day Center’s perinatal mental health intervention program were obtained. This research study was approved by the Research Ethics Committee of the Non-Profit Organization “FAINARETI” (Ref. Number 129/27.12.19). 

### 2.3. Measures

The data derived from:

(*A*) The Medical History (general and mental health) and the socio-demographic data

Before the start of the Day Center’s perinatal health intervention (approximately between the 18th and the 22nd gestation week), a complete medical history of each woman was obtained by the midwife, with an emphasis on information related to woman’s mental health and well-being history and socio-demographic data. This allowed the collection of the necessary information for both the identification of possible pathological mental health symptoms experienced by woman during pregnancy, as well as for the identification of possible risk factors in the woman’s psychosocial history.

(*B*) *Peripartum and Postpartum History*

At 6 weeks after birth, a complete peripartum and postpartum history was obtained by the midwife, including information about the beginning and duration of breastfeeding, both in the hospital and after discharge and about woman’s mental health. Moreover, a supplementary history was obtained at the end of the 1st year after birth, containing information about the baby’s health and behavior, as well as details about the duration of breastfeeding and the introduction of solid foods. A combination of mailed questionnaires and a telephone survey to gather information on maternal and child health indicators are administered to mothers when they are not able to visit the Day Center. Moreover, in the event that a woman continued to breastfeed after the 1st year postpartum, the information on the time of breastfeeding cessation was obtained for all women at 24 months postpartum, either by phone or via email.

(*C*) *PsychometricTools*

In addition to the woman’s medical history taken during pregnancy and at 6 weeks postpartum, the use of psychometric tools enabled the continuation of the detection of pathological mental health symptoms experienced by woman throughout the 1st year postpartum. The psychometric tools were either completed by woman herself and were, then, shortly discussed with the midwife, or were completed by the midwife through an interview. The psychometric tools included: 

(i) *Τhe Edinburgh Postpartum Depression Scale* (EPDS)-Greek version [29,30,31], a widely used screening scale for antepartum and postpartum depression. This tool has been translated and validated for the Greek population by two separate research groups [30,31] and has showed a very high overall internal consistency. Statistical analysis suggested a cut-off point of 11/12, with a sensitivity of 90% and a specificity of 97.2% [30]. Therefore, a total score of 12 or greater has been recommend as an indicator of possible depression [30]. In this study the average alpha coefficient was 0.85.

(ii) *The Patient Health Questionnaire* 9 (PHQ 9) [32]. The PHQ 9 is a 9-item multipurpose instrument for screening, monitoring and measuring the severity of depression symptoms over the previous 14 days. It is the preferred screening tool for depressive symptoms in most primary care settings and obstetrical clinics [33]. It is reported to be specific (>86% at scores of >10) for the identification of people with major depressive disorders [34]. A cutoff of ≥10 was used to define possible depression. In the present study, the average Cronbach’s alpha coefficient was 0.84. 

(iii) *The Perinatal Anxiety Screening Scale* (PASS). It is a 31-item valid, reliable and useful screening tool for the identification of risk of significant anxiety in women in the antenatal and postpartum period [35]. An optimal balance between sensitivity and specificity occurred when caseness was defined by a score of 26 or above. At this cutoff score, 68% of women with a diagnosis of an anxiety disorder were identified [35]. In the present study, Cronbach’s alpha reached 0.94.

### 2.4. Analyses

Absolute (*n*) and relative (*%*) frequencies were used for the description of qualitative variables. *Mean* values and *Standard Deviation* were used for the description of quantitative variables. The data were analyzed using *SPSS* version 22.0. A variety of information on socio-demographic, perinatal, mental health and breastfeeding characteristics was collected. The relationship between peripartum/postpartum factors, any breastfeeding duration and the duration of breastfeeding without giving any formula was assessed through a series of multiple analyses of variance (MANOVA). Also, logistic regression analyses were performed in order to identify factors associated with exclusive breastfeeding at the end of the 6th month postpartum.

The definitions of breastfeeding were determined according to WHO [36] as follows: (a) any breastfeeding was defined as the percentage of infants who received breast milk with or without any other type of food or drink, including breast milk substitutes (non-human milk and formula) and (b) exclusive breastfeeding was defined as the percentage of infants who received only breast milk and no other form of foods or liquids, except for oral rehydration solutions, drops, and syrups (vitamins, minerals, and medicines). In addition, it was considered useful to calculate another breastfeeding indicator, so that the results of this study are comparable with those of the recent Pan-Hellenic research on breastfeeding [27]. Thus, the indicator “Breastfeeding without giving any formula” was calculated, corresponding to the indicator “Breastfeeding without breast-milk substitutes” that was used in the Pan-Hellenic research [27]; the same definition was adopted: “the percentage of infants who receive breast milk with or without any other type of food or drink, except for breast-milk substitutes (non-human milk and formula)” [27]. 

The variable “Women’s mental health status in perinatal period” included (a) women who were mentally healthy (normal mental health), i.e., did not show any pathological mental health symptoms during perinatal period (according to their medical history and/or their scores using psychometric tools), (b) women who had a risk factor in their medical history but did not show any pathological mental health symptoms during the perinatal period (according to their medical history and/or their scores using psychometric tools), and (c) women who suffered from pathological symptoms of mental health disorders (with or without risk factors), as identified by the woman’s medical history and/or through the administration of psychometric tools. Risk factors were defined as mental health disorders in the woman’s family, pathological mental health symptoms in previous periods of her life, bad life events, relationship problems with a partner (domestic violence) and poor socio-economical situation/unemployment. Also, variables concerning the Day Center’s perinatal mental health intervention included the total number of educational and supportive interventions and treatment sessions (antenatally and postpartum), that a woman attended (either alone or with her partner/husband). These sessions were conducted by (a) midwives, (b) mental health professionals and a (c) physical education teacher.

## 3. Results

### 3.1. The Socio-Demographic, Perinatal, Mental Health and Breastfeeding Characteristics

The socio-demographic and perinatal health characteristics of the sample are presented in Table 1. The mean age of the participants was 34.12 (±3.48) years. The majority of them were university graduates (64.9%), were married or had a partner (97%), and almost 70% reported a monthly personal income below 1000 euros (according to Hellenic Statistical Authority, the average annual personal income for 2018 in Greece was 9382 euros). Concerning the perinatal history, almost half of them (49.4%) had given birth vaginally and were satisfied with the procedure (40.3%). The vast majority of women (96.3%) initiated either exclusive breastfeeding (70.7%) or any breastfeeding (25.6%) at the 1st day in the hospital. At the end of the 1st month postpartum, the same percentage of women (96.5%) continued breastfeeding (74.7% exclusive and 21.8% any breastfeeding). Regarding the duration of breastfeeding (a) at the end of the 6th month postpartum: 44.3% of women were breastfeeding exclusively, (b) between 7 to 24 months postpartum: almost 70% of mothers continued any breastfeeding and (c) between 7 to 24 months postpartum: slightly more than half of the mothers (56.1%) continued to breastfeed without giving any formula. Also, slightly less than half of the women (41.7%) were mentally healthy, a similar percentage (45.1%) had some aggravating psychosocial factor in their medical history and a percentage of 12.9% suffered from pathological symptoms of mental health during the perinatal period (Table 1). 

### 3.2. The Midwifery and Psychosocial Intervention Program of the Day Center

In Table 2, data on the sessions of the Day Center’s intervention program, that the participants of this study took part in, are presented. The majority (64.4%) attended, in total, 1 to 15 educational and supportive sessions by a midwife, clinical psychologist or psychiatrist, from the antenatal to the postpartum period, while approximately 1/3 (35.4%) attended a much greater number of sessions, as they received psychological monitoring until the end of the 1st year postpartum. The mean of antenatal group sessions (3-h) by midwives was 7.17 (±2.99), while the mean of midwifery-led supportive individual sessions during perinatal period was higher at 8.22. Almost half of the participants (48.5%) were involved in physical activity sessions antenatally and 25.8% attended psychotherapeutic treatment sessions during the antenatal or postpartum period.

### 3.3. Logistic Regression Analysis and Multivariable Analyses of Variance 

A logistic regression analysis was applied with “Breastfeeding exclusivity at the end of the 6th month postpartum” as the dependent variable (Table 3). Only statistically significant relationships are reported. According to this analysis, for each additional midwife-led session, the chance for exclusive breastfeeding at the end of the 6th month postpartum appeared to increase by 4% (*p* = 0.034). Furthermore, for each additional psychotherapeutic session needed, the chance for exclusive breastfeeding at the end of 6th month appeared to decrease by 18% (*p* = 0.034). Those who did not smoke during pregnancy seemed to have over 0.5 times more chance (55%, *p* = 0.006) for exclusive breastfeeding at the end of the 6th month, while for each day less in NICU, the same chance appeared to increase by around 11% (*p* = 0.002). The exclusive breastfeeding at the 1st day in the hospital (*p* < 0.001), and the exclusive breastfeeding at the end of the 1st month postpartum (*p* < 0.001) seemed to increase the chance for exclusive breastfeeding at the end of the 6th month postpartum by almost 10 and 6 times, respectively (Table 3). 

In Table 4, the results of multivariate analyses of variance (MANOVA) are presented. Only statistically significant relationships are reported. The analyses revealed both any breastfeeding duration and breastfeeding duration without giving any formula to be significantly associated with those participants who conceived naturally (*p* < 0.001 and *p* < 0.001, respectively), did not smoke during pregnancy (*p* < 0.001 and *p* < 0.001, respectively), had a normal delivery (*p* < 0.001, *p* < 0.001, respectively), whose newborn weighed more than 3500 g (*p* = 0.001 and *p* = 0.004, respectively) and whose newborn was not admitted to the NICU (*p* < 0.001 and *p* < 0.001, respectively). The women who had completed master’s degree and/or PhD studies were more likely (*p* = 0.035) to have a longer duration of breastfeeding without giving any formula than the others. The participants who exclusively breastfed their infants at the hospital and at the 1st month postpartum, appeared to have a longer any breastfeeding duration (*p* < 0.001 and *p* < 0.001, respectively) and duration of breastfeeding without giving any formula (*p* < 0.001 and *p* < 0.001, respectively), in relation to those who were only giving formula.

Analyses involving women’s mental health status showed that any breastfeeding duration was significantly associated with the healthy participants (*p* = 0.029), and those who had needed only 1–3 psychotherapeutic sessions (*p* = 0.013) (Table 4). Furthermore, the results revealed an overall tendency of significantly longer any breastfeeding duration (*p* = 0.015) and duration of breastfeeding without giving any formula (*p* = 0.015) for participants who had followed 13 or more midwifery-led supportive sessions (antenatally and postpartum), in relation to those who had attended less than six, or had not participated at all. Finally, the analysis indicates that the participants who had followed seven or more physical activity sessions (antenatally) had a significantly longer any breastfeeding duration (*p* < 0.001) and duration of breastfeeding without giving any formula (*p* < 0.001), in relation to those who had not attended any such session (Table 4).

## 4. Discussion

This study investigates the association between an innovative perinatal health intervention for the care of women, implemented from pregnancy until the end of the 1st year postpartum, on the onset, prevalence, exclusivity and duration of breastfeeding. The innovation of the program was based on both the application of interdisciplinarity (collaboration between midwives and mental health professionals) and on the fact that this intervention put 3 important research assumptions into practice, which are internationally associated with increased initiation and continuation rates of breastfeeding: (a) midwifery-led antenatal education/counselling for the pregnant woman and her partner [17,37,38,39,40], (b) midwifery-led continuous long-term support, counselling and monitoring of breastfeeding during the puerperium and lactation period [40,41,42,43] and (c) early detection of symptoms of mental health disorders and early psychosocial intervention [44,45]. Thus, in this research, breastfeeding is examined in a psychosocial context, in which some of the most important components that are directly related to its promotion and support were covered and satisfied in a timely manner.

The vast majority (96.3%) of participating mothers in the first 24 h after giving birth initiated breastfeeding either exclusively (70.7%) or some of the time (25.6%) and only 3% of them exclusively gave formula. These data coincide with the prevalence rate of breastfeeding (94.2%) during the first 24 h after birth (either exclusively at 65.8% or any breastfeeding at 28.4%), that was found in the most recent Pan-Hellenic study on breastfeeding, conducted in Greece in 2017, in which 549 dyads participated (mother/father and infant) [26,27]. Regarding the following months, the comparison of the results of this research with those of the Pan-Hellenic research of 2017 presents some remarkable findings, which are mentioned below: (a) At the end of the 1st month postpartum: in this study, the percentage of any breastfeeding remains exactly the same (96.5%) as in the 1st day in hospital, while in the Pan-Hellenic research, it appears to have decreased (79.9% versus 94.2% in the first 24 h postpartum) [26]. (b) At the end of the 1st month postpartum: in this study, the percentage of exclusive breastfeeding remains at the same level as in the 1st day after birth (74.7% vs. 70.7%), while in the Pan-Hellenic research, it is decreased (40% vs. 65.8% in the 1st day postpartum) [26]. (c) At the end of the 6th month postpartum (regarding exclusive breastfeeding): in this study, approximately half of the women (44.3%) were breastfeeding exclusively, while the corresponding prevalence in the Pan-Hellenic survey was minimal (0.78%) [26,27]. (d) At the end of the 6th month postpartum (regarding any breastfeeding): in this study, approximately 70% of all women continued any breastfeeding compared with only 45.4% in the Pan-Hellenic survey [26]. (e) At the end of the 6th month postpartum (regarding breastfeeding without giving any formula): in this study, slightly more than half of all women (56.1%) continued breastfeeding without giving any formula compared to only 23.5% in the Pan-Hellenic survey [26]. 

International studies have shown that, although a large percentage of mothers start breastfeeding immediately after giving birth in many countries, over time, most of them stop any and, especially, exclusive breastfeeding, by the end of the 6th month, while the decrease in breastfeeding mothers is observable from the first month after childbirth [46,47,48]. This sad reality is present worldwide and is a worrying phenomenon, as it is known that the benefits of breastfeeding increase with duration and exclusivity [49]. A more optimistic picture of breastfeeding emerges from this study; not only do the vast majority of mothers start breastfeeding (96.3%) and a very high percentage of them begin breastfeeding exclusively (70.7%), but there is a stable exclusive and any breastfeeding process after the 1st month postpartum too. At the same time, there is a larger percentage of mothers, in relation to national and international data [26,27,47,48], who breastfeed exclusively at the end of the 6th month postpartum (44.3%). Also, a very high percentage continues either any breastfeeding (almost 70%) or breastfeeding without giving any formula (56.1%) for a long time (7 to 24 months postpartum). The previous high percentages and encouraging data confirm that 2 of the 3 conditions implemented by midwives during this perinatal health intervention, namely the antenatal education/counselling of prospective parents [17,37,38,39,40,50] and the provision of continuous support to breastfeeding mothers for a long time after childbirth [40,41,42,43], continue to be supportive conditions for the promotion of breastfeeding. According to systematic reviews, such interventions, which begin antenatally and continue throughout the postpartum period, are considered more effective for the duration and exclusivity of breastfeeding than only antenatal or postnatal programs alone [51,52]. 

The above finding is also supported by the results of the statistical analyses of this study. The multivariate analyses of variance (MANOVA) show that mothers who received larger (quantitatively) midwifery support (antenatally and postpartum), had a longer any breastfeeding duration and longer duration of breastfeeding without giving any formula, compared to mothers who did not receive any support or received less support (*p* = 0.015 and *p* = 0.015, respectively). Also, according to the logistic regression analysis, the chance for exclusive breastfeeding at the end of the 6th month postpartum appeared to increase, when the woman received greater (quantitatively) midwifery-led support in the perinatal period (*p* = 0.034). A recent systematic review arrives at the same result; the quality and quantity of midwifery-led breastfeeding support services is associated with an increase in both the duration and exclusivity of breastfeeding [40]. The crucial role of midwives in the promotion of breastfeeding and the importance of the education and support that they provide to breastfeeding mothers have been repeatedly highlighted in the international literature, both by the professionals themselves as well as by the mothers [53,54,55]. 

An additional factor that is closely related to the exclusivity and duration of breastfeeding, and is often not evaluated to the extent that it should be, is the woman’s mental health (antenatally and postpartum) [25,56,57,58,59]. The results of this study show that women who did not experience any symptoms of mental health disorders during pregnancy and up 12 months postpartum and those who did not need to receive long-term counselling/psychotherapy from a mental health professional in the above period had a longer any breastfeeding duration compared to those who showed increased scores on psychometric tools and probably suffered from a mental health disorder, and those who received long-term counselling/psychotherapy (*p* = 0.029 and *p* = 0.013, respectively). Also, the chance for exclusive breastfeeding at the end of the 6th month postpartum appeared to decrease when the mother needed long-term psychotherapy (*p* = 0.034). These results are consistent with a wealth of research that has shown that mothers who experience, antenatally or postpartum, symptoms of mental health disorders, either do not start breastfeeding, stop breastfeeding early, or do not breastfeed exclusively [25,56,59,60]. As it has, characteristically, been mentioned in a systematic review: “early exclusive and non-exclusive breastfeeding cessation is associated with the presence of postpartum depression in all studies published in the last 30 years” [25].

The symptoms of mental health disorders (e.g., anxiety disorder symptoms, depressive symptoms, bipolar disorder symptoms, etc.) are often not diagnosed in time, and as a result, women do not receive the appropriate treatment [61] and they continue to suffer silently. The early detection of symptoms of mental health disorders occurs only in a small percentage of women. According to the literature (a) the detection of anxiety disorder symptoms in the perinatal period is associated with increased difficulties in breastfeeding for the mother, as well as shorter breastfeeding initiation and duration [58,62,63,64,65] and (b) the symptoms of anxiety are often accompanied by depressive symptoms [66,67]. Knowing the above, as well as that the manifestation of depressive symptoms in pregnancy (a) is the best predictor of the occurrence of a mental health disorder postpartum [68,69], (b) was shown to predict early breastfeeding cessation [70,71] and (c) is a strong predictor of a shorter breastfeeding duration [56,57,65], the early detection of symptoms of mental health disorders is very important, as it allows early treatment, in many cases, from the beginning of pregnancy. It is already known that the psychosocial factors that create significant complications in breastfeeding can be modified through interventions and research experience [72]. Thus, screening for mental health disorders for all pregnant women and new mothers and also timely treatment, could probably be related with an improved breastfeeding outcome. Further research on the psychosocial factors that, significantly and systematically, affect the initiation and continuation of exclusive and any breastfeeding is needed, especially the role of early detection and timely treatment of mental health disorders, in order to highlight other aspects of the connection between breastfeeding and maternal mental health in the perinatal period. 

Furthermore, this study significantly correlates specific perinatal factors with exclusive and any breastfeeding duration, and breastfeeding duration without giving any formula. So, smoking during pregnancy (*p* = 0.006) and admission in Neonatal Intensive Care Unit (NICU) (*p* = 0.002) seemed to decrease the chance for exclusive breastfeeding at the end of the 6th month postpartum. In contrast, exclusive breastfeeding at the 1st day (*p* < 0.001) and at the 1st month postpartum (*p* < 0.001) seemed to increase this chance. Also, it is shown that natural conception (*p* < 0.001 and *p* < 0.001, respectively), not smoking during pregnancy (*p* < 0.001 and *p* < 0.001, respectively), vaginal childbirth (*p* < 0.001 and *p* < 0.001, respectively), a birth weight over 3500 kg (*p* = 0.001 and *p* = 0.004, respectively) and the non-admission of the neonate to the NICU (*p* < 0.001 and *p* < 0.001, respectively), correlated with a longer duration of any breastfeeding and a longer duration of breastfeeding without giving any formula. The above correlations are also confirmed by the international literature, according to which, smoking, type of delivery (caesarean section) and admission to the NICU are critical factors that negatively affect the onset, duration and exclusivity of breastfeeding worldwide [73]. In addition, the mother’s higher educational level seems to favor a longer duration of breastfeeding without giving any formula (*p* = 0.035) in this study, as has been documented elsewhere [50]. 

The first limitation of this study is that it was not conducted as a Randomized Controlled Trial (RCT) study. The original design of the study involved the investigation of the course of breastfeeding after the implementation of a perinatal health intervention (offering continuous long-term midwife-led breastfeeding support and psychosocial support) in comparison with the results of a recent study based on a national sample of women. In a RCT study, the comparison would be performed against a random population of pregnant women receiving standard antenatal and postpartum care, so as to determine the effect of the intervention on the course of breastfeeding. Nevertheless, the methodological design that was used led to important findings and allowed the accomplishment of the aim of this study, namely, the investigation of the connection of continuous long-term midwife-led breastfeeding support in combination with psychosocial support with the outcome of breastfeeding. 

Additionally, this study was conducted in a specific primary mental health care setting. Despite the fact that the research sample included every pregnant woman/new mother who attended the Day Center’s perinatal health intervention and met the admission criteria of this study, it is not considered strictly representative of the Greek population, because the participants came mainly from the region of Attica (with a few exceptions). However, considering that this paper examined the results of a breastfeeding intervention that took place in an area where almost 50% of the Greek population resides, we could surely say that its results are of value because they do not concern a small/numerically limited community of women with specific characteristics, but they come from a large proportion of the Greek population, which showed uniformity of ethnicity. Also, we must report that the intervention was implemented on women who had expressed an interest in either being informed/trained in issues related to childbirth and breastfeeding, and/or receiving psychosocial support, and had requested to join the program themselves. So, we must keep in mind that all the women in this sample had a personal motivation and desire to be informed and trained in breastfeeding. 

## 5. Conclusions

This study demonstrated that perinatal midwifery-led education and counselling on breastfeeding and also midwifery-led continuous postpartum support after childbirth and until the 1st year postpartum seem to be the key parts of the intervention strategies that support and promote breastfeeding. In addition, the maternal mental health disorders as a significant factor in the perinatal period and especially the occurrence of pathological symptoms and the increased need for receiving long-term counseling and psychotherapy must be taken seriously into account in designing breastfeeding supporting interventions. Given the significant lasting benefits of breastfeeding for the health of mothers and their infants, knowledge about effective breastfeeding support programs is essential. Further research is considered necessary in order to investigate the usefulness of similar breastfeeding interventions in general or specific/sensitive female populations, especially in countries with low rates of exclusive and/or any breastfeeding. 

## Figures and Tables

**Table 1:** Socio-demographic, Perinatal, Mental Health and Breastfeeding Characteristics.

Socio-Demographic Characteristics	*n/M*	*%/SD*	Range
Age	34.12	3.48	21–50
Education			
High School	189	17.5	
Bachelor’s Degree	701	64.9	
Master’s/PhD	187	17.3	
Total/Missing	1077/3	99.7/0.3	
Marital Status			
Married	907	84.0	
Not Married	27	2.5	
Single Parent Family	16	1.5	
Companionship	130	12.0	
Total/Missing	1080	100.0	
Monthly Income			
0–500 euros	237	22.0	
501–1000 euros	517	47.9	
>1000 euros	305	28.2	
Total/Missing	1059/21	98.1/1.9	
**Perinatal Health Characteristics**			
In Vitro Fertilization (IVF) Pregnancy			
No	992	91.9	
Yes	88	8.1	
Total/Missing	1080	100.0	
Smoking Before Pregnancy			
No	683	63.2	
Yes	395	36.6	
Total/Missing	1078/2	99.8/0.2	
Smoking During Pregnancy			
No	1001	92.7	
Yes	79	7.3	
Total/Missing	1080	100.0	
Type of Delivery			
Vaginal	533	49.4	
C-section	545	50.5	
Total/Missing	1078/3	99.8/0.2	
Delivery Satisfaction			
None at all/Little	95	8.8	
Neither Satisfied nor Dissatisfied	167	15.5	
Mostly Satisfied	435	40.3	
Total/Missing	1080	100.0	
Newborn Weight			
1500–2500	59	5.5	
2500–3500	709	65.6	
>3500	235	21.8	
Total/Missing	1003/77	92.9/7.1	
Admission to Neonatal Intensive Care Unit (NICU)	.89	3.41	
No	907	84.0	
<8 days	107	9.9	
8–15 days	19	1.8	
>15 days	30	2.8	
Total/Missing	1063/17	98.4/1.6	
**Breastfeeding Characteristics**			
Newborn Feeding at the 1st day in hospital			
Exclusive Breastfeeding	764	70.7	
Breastfeeding and Formula	277	25.6	
Formula	32	3.0	
Total/Missing	1080/7	99.4/0.6	
Newborn Feeding at the end of the 1st month postpartum			
Exclusive Breastfeeding	807	74.7	
Breastfeeding and Formula	235	21.8	
Formula	33	3.1	
Total/Missing	1075/5	99.5/0.5	
Exclusive Breastfeeding Duration (at the end of the 6th month postpartum)			
No Breastfeeding	29	2.7	
Non-exclusive Breastfeeding	561	51.9	
Exclusive Breastfeeding	478	44.3	
Total/Missing	1068/12	98.9/1.1	
Any Breastfeeding Duration	8.40	5.62	0–30
No Breastfeeding	29	2.7	
≤6 months	266	24.6	
7–18 months	691	64.0	
19–24 months	61	5.6	
Total/Missing	1047/33	96.9/3.1	
Duration of Breastfeeding Without Any Formula	9.99	5.53	
No Breastfeeding	108	10.0	
≤6 months	333	30.8	
7–18 months	567	52.5	
19–24 months	39	3.6	
Total/Missing	1047/33	96.9/3.1	
**Mental Health Characteristics**			
Women’s Mental Health Status in Perinatal Period			
Normal Mental Health (Healthy)	450	41.7	
Risk Factors in Medical History	487	45.1	
Pathological Symptoms (with or without risk factors)	139	12.9	
Total/Missing	1076/4	99.6/0.4	
Received Psychotropic Medications at perinatal period			
No	1073	99.4	
Yes	6	0.6	
Total/Missing	1079/1	99.9/0.1	

*Note. M*—Mean; *SD*—Standard Deviation; *n*—frequencies; *%*—relative frequencies.

**Table 2:** The Midwifery and Psychosocial Intervention Program of the Day Center.

Characteristics	*n/M*	*%/SD*	Range
Educational, Supportive and Treatment Sessions by Midwives, Clinical Psychologists and Psychiatrists (Antenatally/Postpartum)	15.08	10.06	1–95
1–6	112	10.4	
7–9	189	17.5	
10–12	219	20.3	
13–15	175	16.2	
16–20	191	17.7	
≥21	191	17.7	
Total/Missing	1077/3	99.7/0.3	
Midwife-led Educational & Supportive (3-h) Group Sessions (Antenatally)	7.17	2.99	0–19
1–3	142	13.1	
4–6	220	20.4	
7–9	526	48.7	
≥10	191	17.7	
Total/Missing	1079/1	99.9/0.1	
Midwife-led Educational & Supportive Individual Sessions (Antenatally/Postpartum)	8.22	3.93	1–34
1	39	3.6	
2–3	93	8.6	
4–6	190	17.6	
7–9	354	32.8	
7–12	291	26.9	
≥13	112	10.4	
Total/Missing	1079/1	99.9/0.1	
Physical Activity Sessions (Group/Individual)—Antenatally	5.00	3.86	1–26
0	556	51.5	
1–3	232	21.5	
4–6	141	13.1	
≥7	150	13.9	
Total/Missing	1079/1	99.9/0.1	
Psychotherapeutic Treatment Sessions (Antenatally/Postpartum)	10.58	13.45	1–75
0	802	74.3	
1–3	137	12.7	
4–12	60	5.6	
≥13	81	7.5	
Total/Missing	1080	100.0	

*Note. M*—Mean; *SD*—Standard Deviation; *n*—frequencies; *%*—relative frequencies.

**Table 3:** Logistic Regression Analysis Model with the Dependent Variable “Breastfeeding Exclusivity at the end of the 6th month postpartum”.

Title Independent Variables	*B*	*S.E.*	*p*	*Exp(B)*
Midwife-led Educational & Supportive Individual Sessions (Antenatally/Postpartum)	0.039	0.018	0.034	10.040
Psychotherapeutic Treatment Sessions (Antenatally/Postpartum)	−0.019	0.009	0.034	0.982
Smoking During Pregnancy	−0.799	0.290	0.006	0.450
Admission in NICU	−0.113	0.036	0.002	0.893
Exclusive Breastfeeding at the 1st day in hospital	30.076	0.739	*p* < 0.001	90.600
Exclusive Breastfeeding at the end of the 1st month	10.715	0.249	*p* < 0.001	50.556

*Notes*. Assessment of interpretive power = 0.495; *B* = logistic coefficient; *S.E.* = standard error of estimate; *p* = significance; *Exp(B*) = exponentiated coefficient.

**Table 4:** Multivariate Analyses of Variance of Any Breastfeeding Duration and Breastfeeding Duration without Giving Any Formula.

Independent Variables	Any Breastfeeding Duration		Breastfeeding Duration Without Giving Any Formula					
	*M*	*SD*	*M*	*SD*	*F*	*df*	*p*	*η*²
Education *								
High School			7.58a	6.57	3.35	2	0.035	0.006
Bachelor’s Degree			8.42ab	5.46				
Master’s/PhD			9.09b	5.08				
IVF Pregnancy								
No	10.17	5.52			12.37	1	*p* < 0.001	0.012
Yes	7.98	5.24						
No			8.62	5.63	18.16	1	*p* < 0.001	0.017
Yes			5.93	4.89				
Smoking During Pregnancy								
No	10.22	5.47			25.21	1	*p* < 0.001	0.024
Yes	6.93	5.44						
No			8.62	5.59	21.62	1	*p* < 0.001	0.020
Yes			5.52	5.28				
Type of Delivery								
Vaginal	10.86	5.36			25.60	1	*p* < 0.001	0.024
C-section	9.15	5.58						
Vaginal			9.14	5.60	17.94	1	*p* < 0.001	0.017
C-section			7.68	5.57				
Newborn Weight								
1500–2500	8.42a	5.24			6.94	2	0.001	0.014
2500–3500	9.90a	5.34						
>3500	11.11b	5.59						
1500–2500			6.90a	5.09	5.62	2	0.004	0.011
2500–3500			8.35a	5.41				
>3500			9.41b	6.39				
Admission in NICU								
0 days	10.18a	5.50			7.83	3	*p* < 0.001	0.022
<8 days	9.70a	5.41						
8–15 days	8.89ab	6.77						
>15 days	5.28b	4.26						
0 days			8.56a	5.58	6.34	3	*p* < 0.001	0.018
<8 days			8.06a	5.81				
8–15 days			7.58ab	5.56				
>15 days			4.07b	4.50				
Women’s Mental Health Status in Perinatal Period **								
Normal Mental Health (Healthy)	10.26a	5.41						
Risk Factors in Medical History	10.08ab	5.37			3.56	2	0.029	0.007
Pathological Symptoms (with or without risk factors)	8.83b	6.36						
Psychotherapeutic Treatment Sessions (Antenatally/Postpartum) *								
1–3	10.96a	6.16			3.62	2	0.013	0.10
4–12	9.18ab	5.47						
≥13	8.54b	6.11						
Newborn Feeding at the 1st day in hospital								
Exclusive Breastfeeding	10.94a	5.42			47.22	2	*p* < 0.001	0.083
Breastfeeding and Formula	7.95a	5.02						
Formula	4.71b	5.09						
Exclusive Breastfeeding			9.30a	5.50	40.41	2	*p* < 0.001	0.072
Breastfeeding and Formula			6.46a	5.29				
Formula			3.45b	4.74				
Newborn Feeding at the end of the 1st month postpartum					117.04	2	*p* < 0.001	0.184
Exclusive Breastfeeding	11.28a	5.08						
Breastfeeding and Formula	6.73a	4.93						
Formula	1.88b	3.56			125.31	2	*p* < 0.001	0.194
Exclusive Breastfeeding			9.78a	5.16				
Breastfeeding and Formula			4.68a	4.91				
Formula			1.09b	2.96				
Midwife-led Educational & Supportive Individual Sessions (Antenatally/Postpartum)								
1	8.05a	5.71			3.53	3	0.015	0.010
2–6	9.51ab	5.79						
7–12	10.13ab	5.46						
≥13	10.99b	5.06						
1			5.97a	4.96	3.50	3	0.015	0.010
2–6			8.04ab	5.82				
7–12			8.56ab	5.58				
≥13			9.15b	5.35				
Physical Activity Sessions (Group/Individual)—Antenatally								
0	9.27a	5.68			7.03	3	*p* < 0.001	0.020
1–3	10.46ab	5.55						
4–6	10.66ab	5.16						
≥7	11.29b	4.93						
0			7.71a	5.67	6.10	3	*p* < 0.001	0.017
1–3			8.81ab	5.58				
4–6			9.14ab	5.42				
≥7			9.58b	5.40				

* Statistically insignificant for Any Breastfeeding Duration. ** Statistically insignificant for Breastfeeding Duration Without Giving Any Formula. *Note*. *M*—Mean; *SD*—Standard Deviation; *F* criterion; *df*—degrees of freedom; *p*—statistical significance; η²—eta-squared; Means that share a common index (a/b/c/d) do not differ significantly from each other according to the Scheffé post-hoc criterion.

## Data Availability

Data are contained within the article.
